# Impact of anaemia at discharge following colorectal cancer surgery

**DOI:** 10.1007/s00384-020-03611-0

**Published:** 2020-06-02

**Authors:** Rebecca C. Dru, Nathan J. Curtis, Emma L. Court, Catherine Spencer, Sara El Falaha, Godwin Dennison, Richard Dalton, Andrew Allison, Jonathan Ockrim, Nader K. Francis

**Affiliations:** 1 Department of General Surgery, Yeovil District Hospital, Higher Kingston, Yeovil, Somerset BA21 4AT UK; 2 University Hospitals Bristol NHS Foundation Trust, Marlborough Street, Bristol, BS1 3NU UK; 3 Department of Surgery and Cancer, Imperial College London, Praed Street, London, W2 1NY UK; 4 Division of Surgery and Interventional Science, University College London, London, UK; 5 Northwick Park Institute of Medical Research, Y Block, Northwick Park Hospital, Harrow, HA1 3UJ UK

**Keywords:** Anaemia, Postoperative, Colorectal cancer, Outcomes

## Abstract

**Objectives:**

Preoperative anaemia is common in patients with colorectal cancer and increasingly optimised prior to surgery. Comparably little attention is given to the prevalence and consequences of postoperative anaemia. We aimed to investigate the frequency and short- or long-term impact of anaemia at discharge following colorectal cancer resection.

**Methods:**

A dedicated, prospectively populated database of elective laparoscopic colorectal cancer procedures undertaken with curative intent within a fully implemented ERAS protocol was utilised. The primary endpoint was anaemia at time of discharge (haemoglobin (Hb) < 120 g/L for women and < 135 g/L for men). Patient demographics, tumour characteristics, operative details and postoperative outcomes were captured. Median follow-up was 61 months with overall survival calculated with the Kaplan-Meier log rank method and Cox proportional hazard regression based on anaemia at time of hospital discharge.

**Results:**

A total of 532 patients with median 61-month follow-up were included. 46.4% were anaemic preoperatively (cohort mean Hb 129.4 g/L ± 18.7). Median surgical blood loss was 100 mL (IQR 0–200 mL). Upon discharge, most patients were anaemic (76.6%, Hb 116.3 g/L ± 14, mean 19 g/L ± 11 below lower limit of normal, *p* < 0.001). 16.7% experienced postoperative complications which were associated with lower discharge Hb (112 g/L ± 12 vs. 117 g/L ± 14, *p* = 0.001). Patients discharged anaemic had longer hospital stays (7 [5–11] vs. 6 [5–8], *p* = 0.037). Anaemia at discharge was independently associated with reduced overall survival (82% vs. 70%, *p* = 0.018; HR 1.6 (95% CI 1.04–2.5), *p* = 0.034).

**Conclusion:**

Anaemia at time of discharge following elective laparoscopic colorectal cancer surgery and ERAS care is common with associated negative impacts upon short-term clinical outcomes and long-term overall survival.

**Electronic supplementary material:**

The online version of this article (10.1007/s00384-020-03611-0) contains supplementary material, which is available to authorized users.

## Introduction

Colorectal cancer (CRC) patients are often anaemic at diagnosis and this is commonest with advanced proximal colonic tumours [1–4]. The prevalence of anaemia in this patient group ranges from 30 to 75.8% [1, 4].

The prognostic value of preoperative anaemia in various CRC subgroups has been studied with reported associations between anaemia and adverse outcomes including decreased survival [5–9]. These findings underpin a number of clinical guidelines stating preoperative anaemia should be identified and corrected [10, 11].

Comparatively little attention is given to the prevalence and consequences of postoperative anaemia with no available reports on its impact. Postoperative anaemia may influence functional recovery and overall patient experience including after discharge. A recent international consensus statement on anaemia management after major surgical procedures recommended correction prior to discharge [12] but there is no specific data following colorectal cancer surgery. We therefore aimed to investigate the frequency of postoperative anaemia and any associated short- and long-term implications following colorectal cancer resection.

## Methodology

We performed an observational review of a dedicated prospectively compiled colorectal cancer patient database managed by a specialist information analytical team. Inclusion criteria were patients with biopsy proven colorectal cancer undergoing elective laparoscopic resection with curative intent between 2002 and 2015. Patient demography (age, sex, body mass index (BMI), American Society of Anaesthesiologists (ASA) grade) and histopathologically defined tumour staging data was reviewed. The primary endpoint was postoperative anaemia at time of discharge defined as haemoglobin (Hb) < 120 g/L for women and < 135 g/L for men using the last laboratory measure prior to hospital discharge. All cases began with a laparoscopic approach and were cared for within an established ERAS programme [13–15].

Secondary endpoints included preoperative anaemia (closest Hb measurement prior to surgery), perioperative time (from skin incision to completion of skin closure), total blood loss (volume of blood collected in suction systems and weighed swabs) and conversion to open surgery (inability to complete the dissection laparoscopically including vascular ligation(s) and/or requiring an incision larger than that required to remove the specimen [16, 17]). Postoperative data included length of hospital stay (LoS, number of nights in hospital until discharge to home or care facility as appropriate with the day of surgery designated day zero), major postoperative complications (Clavien-Dindo grade III–V [18]) and readmission (unplanned hospital attendance within 30 days of discharge). All patients entered into a clinical, radiological and endoscopic surveillance follow-up programme for 5 years. Follow-up was defined as time of surgery to last clinical contact or death. This manuscript has been designed in accordance with the STROBE guidelines [19]. Initial creation and anonymised review of the database were approved by the local research ethics and data governance boards.

All data was explored for normality and displayed as mean ± standard deviations unless specified where medians with interquartile range are shown. Mean Hb below lower limit of the sex-specific normal range (LLN) was calculated for anaemic patient groups. *t* test, Mann-Whitney *U* and Kruskal-Wallis tests were used to compare medians from normal and non-normally distributed populations respectively. For categorical data, cross-tabulation and chi-squared testing assessed the difference between groups. Fisher’s exact test was used when appropriate. The Kaplan-Meier log rank method was used to compare overall survival between groups. Cox proportional hazard regression assessed the effect of anaemia at discharge on survival probability simultaneously controlling for clinically relevant factors (age, sex, tumour location, stage, preoperative chemotherapy and postoperative complications). Diagnostics of independent variables confirmed that collinearity was not problematic (variance inflation factor < 2). Results are reported as hazard ratios (HR) with 95% confidence intervals. *p* < 0.05 was considered significant. The data was analysed using SPSS (v26, IBM, USA) and STATA (v8, StataCorp LLC, USA).

## Results

**Fig. 1:**
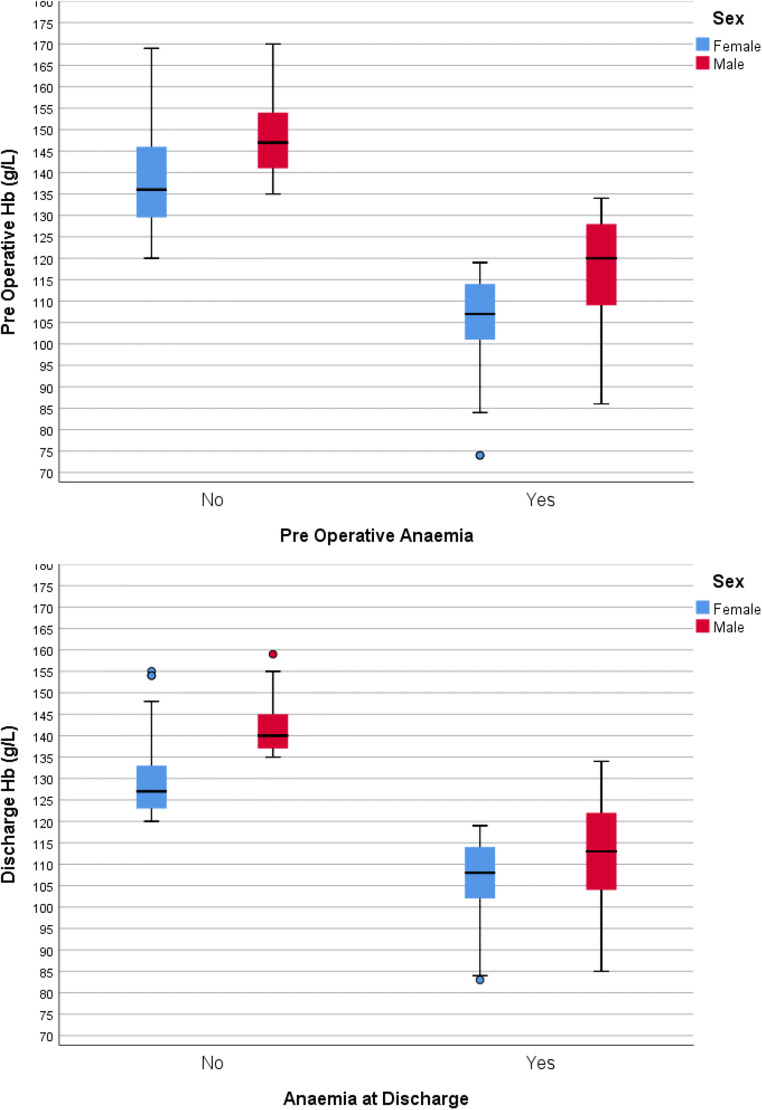
Boxplots displaying haemoglobin values for males and females (g/L). **a** Preoperative values in those with and without anaemic. **b** Corresponding values at time of discharge

A total of 532 patients were included with average age of 70 ± 11 years and BMI 27 ± 5. Three hundred eighty-nine were male. Two hundred eight (39%) had rectal cancer surgery. Two hundred eighty-three patients (55.5%) had stage I–II disease, while 162 patients (31.8%) were stage III (Table 1). 46.4% were anaemic preoperatively (cohort Hb 129.4 g/L ± 18.7, anaemic patients 114 g/L ± 13, 16 g/L ± 11 < LLN Hb, Fig. 1a). Patients with colonic cancer were more likely to be anaemic compared with those with rectal cancers (52% vs. 38% *p* < 0.001; 126.7 g/L ± 19.5 vs. 133.7 ± 16.5, *p* = 0.01, Table 2). Right-sided colon cancers had the lowest preoperative Hb (*p* < 0.001).

**Table 1:** Patient demographics and tumour characteristics

		Mean	Standard deviation	*n*	%
Age (years)		70	11		
Sex	Female			221	41.5
	Male			311	58.5
Body mass index (kg/m^2^)		27	5		
American Society of Anaesthesiologists physical status score	I			60	11.4
	II			337	63.8
	III			128	24.2
	IV			3	0.6
Tumour location	Colon (*n* = 324; right colon 163, transverse colon 44, left-sided colonic 117)				60.9
	Rectum (*n* = 208)				39.1
Tumour stage (histopathologically determined - TNM5th edition)	Benign/pathological complete response to neoadjuvant therapy			35	6.9
	I			114	22.4
	II			169	33.1
	III			162	31.8
	IV			30	5.9

**Table 2:** Frequency and degree of anaemia for different cancer sites. All Hb figures represent g/L. Colonic cancer was more likely to be anaemic with lower Hb compared with rectal cancers (*p* < 0.001). As would be expected, right-sided cancers had the lowest Hb although with relatively small absolute differences (*p* < 0.001)

		Tumour location															
		Left colon				Right colon				Transverse colon				Rectum			
		Count	%	Mean	SD	Count	%	Mean	SD	Count	%	Mean	SD	Count	%	Mean	SD
Preop anaemia	No	61	52.1			73	44.8			21	47.7			130	62.5		
	Yes	56	47.9			90	55.2			23	52.3			78	37.5		
Preop Hb				128	18			125	20			128	20			134	16
Discharge anaemia	No	32	27.6			33	20.2			11	25.0			48	23.2		
	Yes	84	72.4			130	79.8			33	75.0			159	76.8		
Discharge Hb				116	14			114	14			116	13			118	14

A total of 406 patients were anaemic at discharge (76.6%, Hb 116.3 g/L ± 14, Table 3, Fig. 1b). The average preoperative to discharge Hb decrease was 13.6 g/L ± 16.1.

**Table 3:** Haemoglobin values. All figures represent g/L. Negative values represent an increase in Hb between the preoperative and discharge Hb result. Asterisk denotes figures that only include patients anaemic at that timepoint. *LLN* lower limit of normal Hb range

	*n*	Mean	Std. deviation	Anaemic (%)	Minimum	Maximum
Preoperative Hb	532	129.4	18.7	46.4	74	170
*Preoperative Hb below LLN	247	16.3	11.3		1	49
Postoperative Hb	532	113.4	16	80.6	72	157
Discharge Hb	532	116.3	14	76.6	83	159
*Discharge Hb below LLN	406	18.8	11.1		1	50
Preoperative to discharge Hb change	532	13.6	16.1		− 37	45

Median operative time and blood loss were 180 min (IQR 150–250) and 100 mL (IQR 0–200) respectively. A total of 433 procedures were completed laparoscopically with 99 (18%) conversions. Converted patients were more likely to have been anaemic preoperatively (56.6% vs. 44.1%, *p* = 0.025), experience more intraoperative blood loss (225 mL (IQR 100–600) vs. 50 (IQR 0–150), *p* = 0.001), develop more complications (37.4% vs. 12%, *p* = 0.001) and anaemic when discharged (88.9% vs. 73.8%, *p* = 0.001). Overall LoS was 7 days [5–10]. Preoperative anaemia did not alter LoS (7 [5–10] vs. 7 [5–9] days, *p* = 0.063) but those anaemic at discharge had longer hospital stays (7 [5–11] vs. 6 [5–8] days, *p* = 0.037).

Eighty-nine patients (16.7%) developed major postoperative complications. Preoperative anaemia did not alter complication rates (50.6% vs. 45.6%, *p* = 0.392) but patients with complications were more likely to be anaemic at discharge (88.9% vs. 74%, Hb 112 g/L ± 12 vs. 117 g/L ± 14, Hb < LLN 18 ± 14 g/L vs. 11 ± 11, all *p* < 0.001) and have longer LoS (9 [6–18] vs. 7 [5–9] days, *p* = 0.001). Complications were not associated with 5-year survival (HR 1.03 (95% CI 0.7–1.5), *p* = 0.87).

Seventy-four patients (13.9%) were readmitted to hospital but neither preoperative or discharge anaemia was associated (51.4% vs. 45.6%, Hb 129 g/L ± 19 vs. 130 g/L ± 19, *p* = 0.361 and 80.8% vs. 75.9%, 114 g/L ± 16 vs. 117 g/L ± 14, *p* = 0.360 respectively). Patients with postoperative complications were more commonly readmitted (27% vs. 15.1%, *p* = 0.011).

**Fig. 2:**
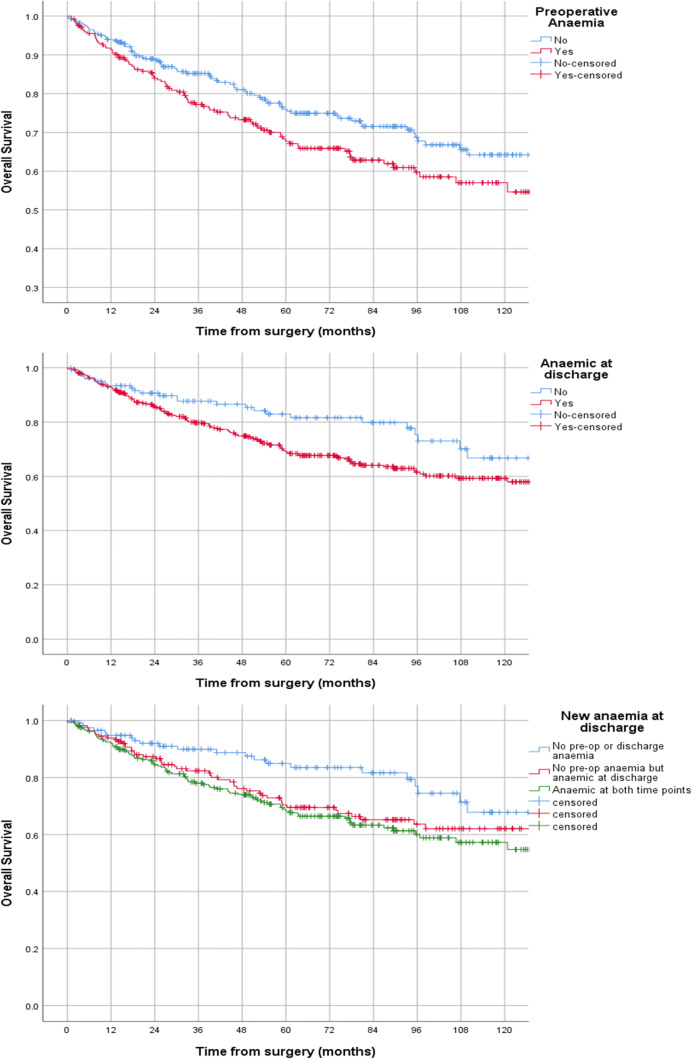
Kaplan-Meier curves displaying overall survival for preoperative anaemia (*p* = 0.027), postoperative anaemia (*p* = 0.018) and those newly anaemic at time of discharge (*p* = 0.018). In all, anaemic is associated with reduced long-term survival

Cohort follow-up was 61 months (IQR 26–93). Tumour stage was not associated with preoperative or discharge Hb levels or anaemia (supplementary Table 1). Five-year overall survival was 74%. Patients anaemic at the time of surgery were seen to have lower 5-year overall survival (76% vs. 67% *p* = 0.027, Fig. 2a) as were those that were anaemic at the time of discharge (82% vs. 70% *p* = 0.018, Fig. 2b). Patients newly anaemic at discharge also had reduced overall survival (83% vs. 70%, *p* = 0.018, Fig. 2c). Anaemia at discharge was independently associated with reduced overall survival (82% vs. 70%, *p* = 0.018; HR 1.6 (95% CI 1.04–2.5), *p* = 0.034). No other covariate was associated with survival on univariate or multivariate analyses.

## Discussion

Preoperative optimisation of anaemia has become well embedded within perioperative protocols but literature on postoperative anaemia and its sequelae remains scarce. Although an international consensus statement on postoperative anaemia management is available [12], the compliance with this guidance and the extent of the problem are unknown. Our study demonstrated the majority of our colorectal cancer patients were discharged anaemic and this impacted short- and long-term outcomes.

With no prior evidence for comparison, we report a potential association between anaemia and lower long-term overall survival. Although the exact mechanism(s) behind this link is unknown, this finding is comparable with CRC reports on preoperative anaemia and overall survival [8]. There is likely to be a wide array of interacting and contributing factors behind these observations including tumour cellular hypoxia and the ability to offer timely adjuvant chemotherapy and the subsequent response [4, 20].

This concerning survival data is of importance given the very high rate of anaemia at discharge consistent with the limited available literature on other major procedures [21]. The precise causes of high postoperative anaemia rates among CRC patients are unclear. A significant proportion had preoperative anaemia which was not corrected prior to surgery and persisted through to discharge. This is particularly likely in the early phase of our cohort prior to our adoption of the current British Society of Haematology guidelines [10] although the rate of postoperative anaemia was higher than preoperative anaemia.

Intraoperative blood loss is another contributing factor to anaemia after surgery, but this was not a major cause in our patients and consistent with contemporary practice data including the lower volume blood loss associated with laparoscopic surgery [22–24]. Conversion to open surgery resulted in higher intraoperative blood loss, postoperative anaemia with implications for postoperative outcomes.

Hospitalisation and postoperative complications are known risk factors for postoperative anaemia due to inadequate nutritional intake, malabsorption, frequent blood sampling and the systemic response to surgical stress [25–27]. This cohort was enrolled within an established ERAS programme. We have previously demonstrated that a significant proportion of patients deviate in more than one element of the postoperative ERAS pathway even in the absence of major complications resulting in longer LoS [28]. It is not clear if postoperative anaemia was a cause or a result from the delayed discharge and prolonged hospitalisation, and further studies are required to specifically address this issue as well as correcting postoperative anaemia and measuring the associated implications.

Traditional ERAS discharge criteria have strong emphasis on functional recovery metrics such as independent mobilisation, resuming oral intake and pain control with adequate analgesia. These discharge criteria could be modified to incorporate checking and correcting anaemia before discharge as this enhances functional recovery and the overall patient experience [29].

Although our pragmatic study is the first to explore anaemia at discharge and its outcomes, it has a number of limitations. Our findings should be interpreted with caution as these observations do not imply causation. Our study generates an interesting hypothesis worthy of further research to study the mechanism of this association and the external validity of these findings. Nevertheless, these findings suggest optimal patient care may involve the correction of anaemia before discharge.

We are unable to control for confounding factors such as comorbidities and the likely differing anaemia management and interventions that could influence our results and were likely to differ across the long study timeframe. The sample size limited meaningful sub-group analysis for relevant areas such as WHO criteria for severity of anaemia [30] and identifying a threshold figure to trigger anaemia correction. Both cancer specific and disease-free survival data is of interest but this was not routinely captured in these patients. Interestingly admission and discharge Hb levels were not associated with tumour stages in our cohort. We included only laparoscopic cases which had strict selection criteria particularly in the early phase of the study timeframe which may explain the relatively low number of advanced tumours in this cohort. Additionally, full data on the nature and severity of complications was incomplete particularly for the earliest patients and was not obtainable. Finally, our study design prevented capture of postdischarge patient centred metrics such as functional recovery, quality of life and return to work which remain under-reported in ERAS research. Further research is now indicated to record and assess corrective actions of correcting anaemia at discharge and their impact on functional and oncological outcomes.

## Conclusion

Anaemia at time of discharge following elective laparoscopic colorectal cancer surgery is commonplace with associations upon short-term clinical outcomes and long-term overall survival. Correction of anaemia before leaving hospital may represent beneficial discharge criteria for future ERAS protocols.

## Electronic supplementary material

ESM 1 (DOCX 22 kb)
