# Supplementary material for: Impact of anaemia at discharge following colorectal cancer surgery
Source: Int J Colorectal Dis. 2020 Jun 2;35(9):1769–76. doi: 10.1007/s00384-020-03611-0 (PMC7415032; doi:10.1007/s00384-020-03611-0)
Supplement: Supplementary file 1 — (DOCX 22 kb) [file 384_2020_3611_MOESM1_ESM.docx]

Supplementary table 1: Effect of tumour stage on pre-operative and discharge Hb levels. Stage 0 includes pathological complete response to neoadjuvant chemoradiotherapy and unexpectedly benign histology results.

|  | | Preoperative Hb | | Preoperative Anaemia | | | | Discharge Hb | | Discharge Anaemia | | | |
| --- | --- | --- | --- | --- | --- | --- | --- | --- | --- | --- | --- | --- | --- |
|  |  | Mean | SD | No | | Yes | | Mean | SD | No | | Yes | |
|  |  |  |  | Count | % | Count | % |  |  | Count | % | Count | % |
| Stage | 0 | 136 | 19 | 23 | 65.7% | 12 | 34.3% | 116 | 13 | 9 | 25.7% | 26 | 74.3% |
|  | 1 | 128 | 20 | 53 | 46.5% | 61 | 53.5% | 117 | 15 | 29 | 25.9% | 83 | 74.1% |
|  | 2 | 128 | 18 | 90 | 53.3% | 79 | 46.7% | 115 | 13 | 35 | 20.7% | 134 | 79.3% |
|  | 3 | 130 | 19 | 89 | 54.9% | 73 | 45.1% | 117 | 14 | 42 | 25.9% | 120 | 74.1% |
|  | 4 | 129 | 17 | 16 | 53.3% | 14 | 46.7% | 113 | 13 | 5 | 16.7% | 25 | 83.3% |
